# Teachers’ Conflict-Inducing Attitudes and Their Repercussions on Students’ Psychological Health and Learning Outcomes

**DOI:** 10.3390/ijerph16142534

**Published:** 2019-07-16

**Authors:** Muhammad Rashid Ali, Badar Nadeem Ashraf, Chuanmin Shuai

**Affiliations:** 1 School of Economics and Management, China University of Geosciences (Wuhan), Wuhan 430074, China; 2 School of Finance, Jiangxi University of Finance and Economics, Nanchang 330013, China

**Keywords:** incivility, ethnic discrimination, university resources, students’ educational outcomes, students’ psychological health

## Abstract

This paper studied the causes and effects of negative teacher–student relationships on students’ psychological health and educational outcomes, primarily due to negative teacher–teacher interactions. Survey data were collected from 130 faculty members and 746 students of 10 higher educational institutions located in different cities of the Punjab province of Pakistan. Path analysis was used to estimate results. The findings revealed that incivility among faculty members and higher discontent with university resources generates a conflict-inducing attitude in faculty members, which subsequently creates negative behavior in teachers towards students. It was further observed that hostile attitudes of faculty members towards students adversely affects the psychological health and educational outcomes of students at universities. These findings suggest that students’ learning processes can be improved by controlling negative teacher–teacher interactions, which has important implications for institutions of higher learning.

## 1. Introduction

Knowledge is the key to success in every field of life, and the process of seeking knowledge spans from the cradle to the grave. Across the world, the education sector has always been the foremost medium for human learning. The learning process largely depends on the effectiveness of the education sector. In this context, the focus of educational policymakers is primarily to identify the factors that can play imperative roles in the learning outcomes of students. Effective teaching and learning environments have a direct relationship with the mental and relational behavior of students. Although numerous studies have focused on the constructive behavior of educators, the literature regarding destructive demeanors of educators is scarce. Additionally, some studies have found that some teachers are professionally unskilled, and, hence, use outdated teaching techniques [1,2,3]. Regrettably, many factors, which include but are not limited to teachers’ low remuneration, non-comprehension, misbehavior, authoritative behavior, excessive workload, lack of in-service training [4,5], gender bias among teachers [6], and university culture [7] can escalate the hostile attitudes of a teaching faculty. Destructive behavior further deteriorates the situation and negatively influences the interactions between the teachers and students. Conflict-inducing attitudes of teachers have a negative impact on students, and can hamper students’ learning processes and their psychological well-being in educational institutions [8,9,10].

Although some studies have taken into consideration the impact of faculty members’ rudeness, discrimination, and university resources on teacher–student relationships, but the impact of these factors on students’ psychological health and learning outcomes has not yet been studied. This research is among the first studies to focus on the mental altercations of students, which will distinguish this research from former studies. In this study, we have suggested a set of variables including discrimination among faculty members, faculty members’ incivility to each other, and university resources, which induce an attitude of conflict among faculty members and consequently adversely affect students’ learning and psychological well-being.

Contemporary studies have found that employee incivility, discrimination, and higher discontent with organizational resources can result in conflict-inducing attitudes [8,11,12,13,14,15,16,17]. Conversely, some authors [8,9,18,19,20] have stated that conflict-inducing attitudes in the education sector affect the learning outcomes and psychological traits of students.

To carry out the analysis, we collected data from ten high-ranked educational institutions located in different cities of the Punjab province of Pakistan. Collecting data from both the teachers and students simultaneously was able to better reflect the interconnected factors that can impact students’ educational and psychological outcomes. We solicited questionnaires from teachers to inquire about faculty members’ discriminatory attitudes towards each other, misbehavior among faculty members, and the discontent with university resources. We also asked students about the treatment of teachers, incivility, demeanor, students’ educational outcomes, and their psychological complaints. The support of the suggested model in our study will give an insight to managers about the results of adverse interactions, and it will further illuminate the characteristics of numerous teacher-related and student-related aspects, which are the primary cause of these detrimental outcomes.

## 2. Conceptual Framework

**Figure 1:**
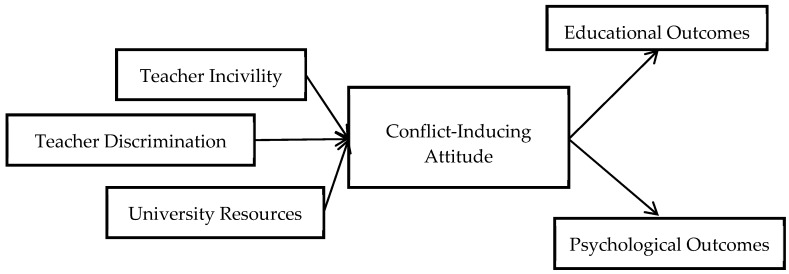
Conceptual model of this study.

The conceptual model of this study is illustrated in Figure 1, which explains the links between the different variables. The first part of the model describes the factors which negatively impact the morale of the teachers, incorporating negative interactions with co-workers, discriminatory behavior amongst colleagues, and the lack of university resources. This causes a conflict-inducing attitude in teachers towards students, and can also put to halt the lecture preparation, grading of assignments and exams, and examination question setting. In the second part, the model describes how adverse effects on these activities may negatively impact students learning and psychological well-being.

Incivility among co-workers is a reason for interpersonal conflict [12,21], which is caused by dissimilar personality traits. These include hostility, mistreatment, mistrust, and inappropriate gestures. Some studies have argued that workplace discrimination among employees could cause indifferent attitudes and strained relationships [16]. Additionally, Sommet, Darnon, Mugny, Quiamzade, Pulfrey, Dompnier, and Butera [14] and Rahim [13] have suggested that strained relationships have a negative impact on social interactions, leading to a situation of conflict. Generally, the likelihood of strained relationships among co-workers is higher at the earlier stages of employment [11,22]. Employees can also be discriminated against based on their specific ethnicity or gender.

Finally, the overall climate of an organization might be a cause of frequent conflicts among employees. For instance, employees who have excessive workloads and face structural problems in routine tasks are more likely to develop a conflicting attitude [15]. Moreover, resource scarcities also have an adverse effect on the performance of teachers. For example, Shoulders and Krei [23] found that the number of hours spent on teachers’ professional development has a positive impact on the teachers’ ability to engage students. Some authors [8,18,19] have also found that conflict-inducing attitudes in the education sector affect learning outcomes and the psychological well-being of students.

## 3. Theoretical Underpinning of the Study

The theoretical underpinning of our hypotheses for this study is adapted from the attitude, behavior, and contradiction ABC framework of Galtung [24] and affective event theory (AET) by Weiss and Cropanzano [25]. AET is based on how individuals execute and react; these reactions are based on emotional attitudes, interactions, and deviant behavior. AET explains the effect of the motivational and emotional aspects that affect mood and states of concentration for positive or negative outcomes [25,26]. It elucidates that the attitude and emotions in the workplace play a significant role in people’s responding to situation either destructively or constructively, and that these attitudes and emotions can be seen through people’s behavior or actions [26,27].

Alola, Avci, and Ozturen [26], Härtel et al. [28], Carlson et al. [29], Shaw [30] and Carlson et al. [31] applied AET to argue that the effects of emotion and motivational content on employee temperament can lead to enhancement of employee job performance, while the negative attitude hampering the motivational drive that will result in deviant behavior leading to adverse impact on their working activities.

The ABC paradigm is a triangle (three mutually interdepending facets) ―attitude, behavior, and contradiction―for exploring the causes and effects of conflict [24]. The ABC framework states that conflict is a contradictory situation, where two or more individuals express an aggressive attitude towards each other for their dissenting interests [24,32,33]. In the workplace, a contradiction occurs when two or more organizational members of the same or different hierarchical levels demonstrate a disagreement or inappropriate behavior [34]. When people work together, conflict is inevitable. Lumby et al. [35] state, “… the very nature of education and schooling generates conflict.” Shemyakina [36] and Bertoni et al. [37] stated that conflicts emerge in many aspects of human life, including in the most respected organizations, such as educational institutes. One serious side-effect of conflict in an educational institution is stress; stressed workers are less satisfied with their job, lose temper and patience, change in behavior, and experience anxiety. Anger and anxiety can reduce employee morale and constructive attitudes.

## 4. Factors That Tend to Lead to Conflict-Inducing Attitudes

### 4.1. Incivility

Incivility is defined as discourteous or inappropriate behavior, whether direct or indirect, oral or corporal, from one or more individuals to harm others at the workplace [38,39,40,41,42]. Accurate statements might spawn a negative behavior when they are articulated in a violent, condemning, or inconsistent way [43,44,45]. Short or frequent interactions between colleagues may originate conflicting behavior. For example, brief discussions can create confusion and reinforce the troubles between individuals [46,47]. By contrast, more frequent interactions can create misunderstanding and subsequent misbehavior [21]. For instance, when individuals interact frequently, they may exchange harsh words, and, afterwards, tend to ignore each other [48]. Individuals may also accuse others because of transgression in facial expressions, which leads to a conflict-inducing attitude [49,50]. Alshehry et al. [51] found that uncivil behavior of supervisors towards their subordinates negatively affects their professional lives, and they, in turn, tend to behave rudely with their subordinates lower in the organizational hierarchy. Cortina et al. [39] also presented evidence that incivility reduces employees’ productivity by adversely affecting their psychological health. Thus, uncivil behaviors demoralizes teachers, reduce motivation for a good teaching environment, and hence causes negative interactions with students.

**Hypothesis 1** **(H1):** *Incivility among faculty members causes conflict-inducing attitudes in teachers.*

### 4.2. Discrimination

When someone is treated unfavorably due to their gender, religion, ethnicity, or political affiliation, the phenomenon is termed discrimination [52]. Workplace discrimination promotes favoritism, domination, and power [53]. It hurts emotions and generates discomfort in some employees [16]. According to Nafees et al. [54], teacher to teacher and teacher to head of the department relationships, due to gender and ethnicity bias, lead to asymmetrical workloads where favored employees avoid tasks while others are overburdened. Such asymmetrical workload has a direct influence on teachers’ attitude towards students and teaching performance. Likewise, Abbas et al. [55] established that gender-based discrimination and preferential treatment for some individuals in promotions and facilities negatively affected other employees’ performance in telecommunication organizations. Relentless gender discrimination ultimately results in the resignation of sufferers, even in the education sector [56,57,58].

Based on this discussion, we draw the following hypothesis:

**Hypothesis 2** **(H2):** *Gender and ethnic discrimination among faculty members causes conflict-inducing attitude in teaching faculties.*

### 4.3. University Resources

Though governments allocate a major portion of their budgets to meet the needs of the education sector, the scarcity of physical and human capital is still a major concern for higher educational institutions. The shortage of resources leads to low salaries of teachers, excessive workload [15,59], or the unavailability of general physical resources in the school [60,61]. Low remuneration and work overburdening hamper employees’ motivation to effectively and efficiently engage in teaching tasks. Likewise, the lack of equipment, such as computers and projectors, and of office space, leads to negative teacher to teacher interactions. As a result, the scarcity of physical and human capital in universities causes conflicts among staff members and adversely affects their performance [62]. For instance, Barsky [63] concluded that a shortage of physical resources reduces employees’ morale for positive interactions with other subordinates. In a similar vein, some authors have found that the school environment is a significant factor for the destructive behavior of the teaching faculty [64,65]. Based on this discussion, we draw the following hypothesis:

**Hypothesis 3** **(H3):** *Higher discontent with university resources causes conflict-inducing attitudes in teachers.*

### 4.4. Effects of Teachers’ Destructive Attitudes on Students

The way in which two or more people relate and behave to each other is defined as a relationship. Teachers’ relationships with their students are a key factor in facilitating or hindering students’ learning and psychological outcomes [66,67,68], and have been researched for decades [61,69,70,71]. The constructs that lead to constructive teacher–student relationships are gratification, respect, cooperation, power, acknowledgment, own sustenance, and societal reception [72,73,74]. The deficiency of any of these characteristics may harm the teacher–student relationship.

Recently, both positive [9,75,76,77] and negative [9,10,20,78] interactions among teachers and students have been studied. Positive interactions lead to improved psychological health and learning performance, while strained relationships yield the opposite [13,14,68]. The negative attitudes of teachers worsen their instructional performance, especially in science-related courses [79]. Teachers’ uncivil behavior demotivates students, causes an unpleasant learning environment, limits students’ course choices [66], and thus negatively affects the students learning [3]. Additionally, discrimination generates stress and a feeling of severance in some faculty members, worsens their teaching ability, and consequently constrains students’ striving for innovation and positive results [80].

Though violence and anger are the pinnacles of uncivil behavior, yelling at others, useless arguments, and deliberate insults also have an adverse psychological effect [10,18,20,81]. The destructive behavior of teachers may result in psychological problems and depression in students [68,82,83,84,85,86]. Likewise, aggressive and violent behavior of teachers not only adversely affects students’ psychological health but also their interactions with other students [87,88].

Based on the above discussion, we state the following two hypotheses regarding the impact of misbehavior on students’ performance and psychological outcomes.

**Hypothesis 4** **(H4):** *Teachers’ negative behavior towards students adversely affects students’ educational learning.*

**Hypothesis 5** **(H5):** *Teachers’ negative behavior towards students adversely affects students’ psychological outcomes.*

## 5. Methods

### 5.1. Measure of Constructs Contained in the Model

There are three exogenous variables: Teacher Incivility (TI), Teacher Discrimination (TD), and University Resources (UR). Students’ Educational Outcomes (SEO) and Psychological Outcomes (PSYO) are the exclusive endogenous dependent variables. Conflict-Inducing Attitude (CIAT) towards students is used as both a dependent and independent variable [8,17,39].

#### 5.1.1. Teacher Incivility

We used the reliable and valid Workplace Incivility Scale (WIS; Cortina et al. [39]). We measured uncivil behavior among faculty members, and between the head of the department and the lower ranked faculty members. A questionnaire with modification had five items. Responses were ranked from 1 = ‘rarely’ to 5 = ‘very often,’ where high scores reflect higher incivility and vice versa.

#### 5.1.2. Teacher Discrimination

To measure the level of discrimination between the colleagues, we used the scale developed by Fox and Stallworth [89]. We had four items with modification. Responses were scaled on a five point Likert scale (1 = ‘strongly disagree’ and 5 = ‘strongly agree’) where higher values represent a higher level of discrimination by ethnicity and gender and vice versa.

#### 5.1.3. University Resources

University resources were measured with two indicators: individuals’ social support (promotion, salary, and incentives) and physical resources (organizational structure and availability of facilities). We used four items from Anderson [90], and modified them to measure university resources. Responses ranged from (1 = ‘strongly disagree’) to (5 = ‘strongly agree’), where higher values of each indicator show a higher level of discontent with university resources and vice versa.

#### 5.1.4. Conflict-Inducing Attitude, Educational Outcomes, and Psychological Outcomes

We used two reliable and valid scales (TMC, Teachers Misbehavior Checklist, and ES, Effect Scale) developed and used by [2,8,87,88] to measure the constructs related to students’ perception. To assess conflict-inducing attitude towards students, eight items were used. Similarly, to analyze the educational effects, five items were used. Responses ranged from 1 to 5, where higher values reflect more uncivil behavior towards students and weak education outcomes. Finally, four items were used to assess psychological effects on students. Again, responses ranked from 1 (strongly disagree) to 5 (strongly agree), where higher values represent more psychological complaints from students.

### 5.2. Data Collection and Respondents’ Demographic Profiles

Data were collected from ten universities located in different cities of the Punjab province of Pakistan. The identities of survey participants were kept confidential. We used two-stage cluster sampling: First, from each selected university, three classes were chosen for the survey. Second, for each class, we selected four to five teachers. With the permission of related heads of departments, all students of a class and related teachers were contacted to fill the questionnaires. Out of the total selected teachers, 87% agreed to participate in the survey. Questionnaires were distributed and respondents were given two to three hours to finish the survey. All efforts were made to ensure the independence of respondents. Finally, we succeeded in collecting data from 130 teachers and 746 students. The survey was conducted in the summer of 2018, and the whole data collection process took almost three months. All the students from the nominated classes were surveyed. They were assured that teachers would not have access to their responses. Each student was asked to assess a specific teacher who was randomly assigned to them. We chose students who were in at least the second year of their university study. In this way, each selected teacher was evaluated by five to ten students. We took an average of students’ responses to get one value for a specific teacher.

The teacher sample included 93 men (71%) and 37 women (29%). A total 41% of teachers were between 25 and 44 years old, and 47.99% were between 25 and 34 years. In the teacher sample, 57% of teachers had an M.Phil. qualification, while 43% held a Ph.D. degree.

The student sample contained 343 male (45.97%) and 403 female (54.03%) students. Ages of most students ranged between 15 and 25 years. The student sample included 447 bachelor students, 226 Masters students, and 73 M.Phil. students.

## 6. Results and Discussion

### 6.1. Descriptive Statistics

Table 1 reports the demographic profiles of both teachers and students who participated in the survey.

### 6.2. Construct Validity

To evaluate the constructs, confirmatory factor analysis (CFA) was conducted to test the fitness of our overall measurement model with the data. To test the relevant reliability, composite reliability and Cronbach alphas were calculated. Factor loading and average variance extracted was checked to assess the discriminate and convergent validity.

As shown in Table 2 and Table 3, the alpha (“α”) values for all components ranged between 0.70 and 0.84. Factor loading on each component was above the threshold value of 0.70, as recommended by Reference [91]. Values of composite reliability exceeded the standard value of 0.70 and fell in the range of 0.82 to 0.93. Standardized factor loading of all items ranged from 0.68 to 0.94, and was significant at a 0.01 level of significance. However, four elements had lower values in factor loading, which was acceptable because Ertz et al. [92] stated that if the values of the factor loading did not affect the overall results, then these values are enough for analysis. Convergent validity fulfilled the overall requirements. However, we excluded two items because their factor loading was less than 0.60, which was not adequate for analysis. The average variance extracted (AVE) values were well above the recommended threshold level of 50% [93] and fell in the range of 0.54 to 0.73, which showed that the maximum variance was interpreted with constructs. We tested for discriminant validity by comparing the square roots of the AVE of each construct (in the diagonal elements in Table 3 and Table 4) with the correlation coefficients across all theoretically related constructs (in the off-diagonal element in Table 3 and Table 4) Fornell and Larcker [94].

The fit indices of the measurement model, (*χ*^2^ = 8.12, *df* = 3, *χ*^2^/*df* = 2.70, goodness of fit index (GFI) = 0.99, adjusted goodness of fit index (AGFI) = 0.93, comparative fit index (CFI) = 0.95, normed fit index (NFI) = 0.91 non-normed fit index (NNFI) = 0.97, root mean square error of approximation, (RMSEA) = 0.027) were proven to have a good fit with the data.

Six constructs were involved in the research. The statistical results predicted that the overall fitness of the model was satisfactory. After verifying the measurement models, correlation and path coefficients were carried out using Lisrel 8.80 (Scientific Software International, Inc., Chicago, IL, USA) to discover the relationships between constructs.

### 6.3. Hypothesis Testing

Table 4 reports the means, standard deviations, and pair-wise correlations for the main variables. Pairwise correlation values in Table 4 indicate that negative behavior, discrimination, university resources, and conflict-inducing attitude all have positive associations with students’ educational outcomes and psychological outcomes. Together, the results of correlation analysis support our hypotheses.

**Figure 2:**
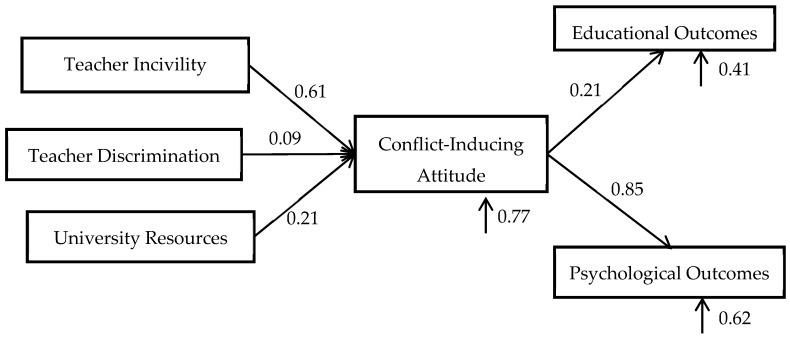
Tested model in our study.

Next, we carried out structural equation modeling (SEM) by using a recursive path analysis technique to test the hypothesized relationships between variables. As shown in Figure 2, we estimated path coefficient results for each relationship.

The hypotheses (H1–H5) were tested with the significance levels of the variables (Table 5). As anticipated, the teachers with conflict-inducing attitude who demonstrated incivility, discrimination, or diminutive university resources, had detrimental effects on students’ psychological health and learning outcomes.

The incivility variable had a positive and significant association with conflict-inducing attitude (*β* = 0.61, *p* < 0.001). This confirms our H1: that incivility among faculty members causes conflict-inducing attitudes.

The evidence for H2 was not robust, as the discrimination variable was insignificant with conflict-inducing attitude variable (*β* = 0.09, *p* = 0.15). This result suggests that discrimination did not generate conflict-inducing attitudes in our sample.

The university resources variable was also positive and significant (*β* = 0.21, *p* < 0.001), showing that higher discontent with university resources caused conflict-inducing attitudes in teachers, which is consistent with our H3.

Next, we report the results where students’ educational and psychological outcomes were used as the dependent variables. As shown in Table 5, the conflict-inducing attitude variable was positively and significantly correlated with the students’ educational outcome variable (*β* = 0.12, *p* < 0.005). Because higher values of the students’ educational outcome variable represent weak performance, these results confirm our H4: that teachers’ negative behavior towards students adversely affects students’ educational learning.

Similar results were observed for psychological outcomes (*β* = 0.85, *p* < 0.001) confirming our H5: that teachers’ negative behavior towards students adversely affects students’ psychological outcomes.

The corollary is that the results confirm the conjecture that our three main variables can affect students’ educational learning and can create psychological complaints through the channel of conflict-inducing attitudes in universities of the Punjab province of Pakistan.

## 7. Discussion

We found evidence that the conflict-inducing attitudes of faculty members adversely affect students’ educational outcomes and psychological health.

Specifically, the result of incivility (0.61) suggests that higher uncivil behavior with the head of department or coworkers strengthens conflict-inducing attitudes in a teaching faculty. Rude behavior is intolerable, even among educated individuals, and not only causes, but also strengthens interpersonal conflict among colleagues [95]. Aggressive behavior leads to rigidity in relationships and bolsters conflict-inducing attitudes [96]. Uncivil behavior reduces morale, performance, and the effectiveness of teacher–student relationships. These findings are consistent with the studies [17,97,98] that have argued that uncivil behavior with colleagues adversely affects employee performance. In an educational setting, it generates problems among teachers and diverts their attention from student-related duties. Teacher-to-teacher incivility reduces discipline in teaching and has the most critical influence on teachers’ morale, attitude, behavior, and relationships with subordinates.

Our result for discrimination was not significant, which suggests that discrimination was less of a concern in our sample. This result might have occurred due to the specific institutional setting of our dataset. We collected the data from the same province, and, by and large, our respondents were of the same ethnicity. This result is also consistent with the findings of Bibi and Karim [17], who did not find gender-discrimination-related issues in the same sample area.

The university resources (0.21) have a direct effect on teacher–student relationships. The scarcity of physical and social resources, which includes the absence of autonomy, low wages, or excessive work-load, creates problems and leads to a frustrated and conflict-inducing attitude in faculty members. Here, we contribute to the previous literature which finds that resources are a basic determinant of a good or bad teaching environment [8,99,100].

Our model suggests that negative teacher–student interactions always generate adverse educational (0.21) and psychological (0.85) outcomes. Focused personality arguments, quarrels over inflexible things, and illegal personal purposes [101] produce fear, distrust, and anger. Hargreaves [73] and Malm [102] argue that mistreatment between individuals seemed to be an exception rather than the norm, and the majority of people remain in silent rivalry. We find that negative interactions, irrespective of their occurrence rate, adversely affect performance. Our findings are comparable with the results of Sava [8] and Stephan and Stephan [103], who concluded that the existence of secrecy, snooping, and inequality had a profound effect on students’ learning. Similarly, Gorham and Christophel [104] argued that 43% of the factors which discourage students’ learning originate from teachers’ behavior. We complement Murray-Harvey and Slee [87], in that they also found out that higher psychological problems in students originate because of teachers’ misbehavior towards them.

## 8. Conclusions

With the survey data collected from teachers and students of 10 universities in the Punjab province of Pakistan, this study explored the causes of conflict-inducing attitudes, which negatively affect students’ educational and psychological outcomes.

Overall, the study revealed that incivility among faculty members and the scarcity of university resources lead to conflict-inducing attitudes in faculty members. Further, teachers’ uncivil behavior to each other and higher discontent with university resources are associated with negative teacher–student interactions, which adversely affect students’ educational and psychological outcomes.

### 8.1. Implications

This study provides important managerial implications. Firstly, managers should monitor the relational responses of teachers. Since our results showed that incivility among faculty members adversely affects their performance, managers should understand and address factors that may create incivility among teaching staff. Strict rules, such as demotion or termination, should be implemented to control these factors.

Secondly, our results indicated that the scarcity of university resources generates a significant, positive influence on teachers’ misbehavior, which implies that educational institutions need to improve salary structure, power imbalances between teachers, deficiency in resources, and to distribute the workload for the enhancement of students’ learning processes. Focusing on such improvement could make educational outcomes more achievable and create a milieu for working.

Thirdly, our study specified that leaders should pay attention to the consequences of conflict, and attempt to abolish the conflict. People frequently engage in detrimental misbehavior. Although it is impossible to eliminate negative teacher–student interactions, an attempt can be made to control teachers’ repetitive aggressive attitudes towards students. Repeated misbehavior has more adverse physical violence and emotional consequences for students [105].

Finally, strategies can be framed and teacher training programs can be arranged to educate teachers to better handle conflict-inducing situations and attitudes.

### 8.2. Limitations and Future Research

One limitation of our study is that we considered only organizational factors for conflict-inducing attitudes. However, personal factors can also be a reason for conflict-inducing attitudes [106], for which we did not control in our model.

Future studies may consider additional factors, such as management policies, which are likely to moderate the relationships between conflict-inducing attitudes and students’ learning outcomes. A study of this kind would help to understand how to devise organizational policies that are effective in controlling teachers’ behavior.

## Figures and Tables

**Table 1:** Demographic information of the respondent.

Profile & Category	Teachers Perspective		Students Perspective	
	No. of Respondents	Percentage of Sample	No. of Respondents	Percentage of Sample
Gender				
Male	93	70.99%	343	45.97%
Female	37	29.01%	403	54.03%
Academic Qualification		,		
Bachelors			447	59.92%
Masters			226	30.29%
MS/M.Phil.	76	57%	73	9.79%
Ph.D.	54	43%		
Age (Years)				
25–34 & 15–25, respectively	52	40.62%	598	80.20%
35–44 & 26–35, respectively	62	47.99%	130	17.40%
45–54 & 36–45, respectively	12	9.54%	13	1.80%
55 and above & 46–55, respectively	04	1.85%	05	0.05%

**Table 2:** Confirmatory factor analysis, teachers’ perspective.

Constructs		Measurement Items	SFL
**During the PAST YEAR, were you ever in a situation in which any of your co-workers or supervisors…?**			
Teacher Incivility	TI1	Paid little attention to your statements or showed little interest in your opinions.	0.68
Cronbach’s alpha (“α”) = 0.77	TI2	Colleagues gave me hostile looks, stares, or sneers.	0.81
CR = 0.892	TI3	Addressed you in unprofessional terms, either publicly or privately.	0.78
AVE = 0.564	TI4	Made insulting or disrespectful remarks about you.	0.77
	TI5	Ignored me or failed to speak to me (e.g., gave me “the silent treatment”).	0.77
**During the PAST YEAR, were you ever in a situation in which, due to gender or ethnicity, any of your co-workers or supervisors…?**			
Teacher Discrimination	TD1	Discrimination (due to gender and ethnicity) was a reason for conflict.	0.75
Cronbach’s alpha (“α”) = 0.707	TD2 *	Accused you of incompetence.	0.52
CR = 0.827	TD3	Doubted your judgment on a matter over which you had responsibility	0.80
AVE = 0.546	TD4	Rated me lower than I deserved on an evaluation.	0.68
	TD5	Ignored or excluded me from social and professional comrades.	0.72
**During the PAST YEAR, were you ever in a situation of scarce resources in your university?**			
University Resources	UR1	I think that lack of facilities creates conflict, due to inadequate financial resources.	0.87
Cronbach’s alpha (“α”) = 0.761	UR2	Incentives and salary are less than I deserve.	0.77
CR = 0.845	UR3	Poor physical resources of my university affect working conditions badly.	0.68
AVE = 0.579	UR4	I have been pressured to use only available resources.	0.71

* Items were dropped; CR, Composite Reliability; AVE, Average Variance Extracted; TI, Teacher Incivility; TD, Teacher Discrimination; UR, University Resources; SFL, Standardized Factor Loading.

**Table 3:** Confirmatory factor analysis, students’ perspective.

Constructs		Measurement Items	SFL
**During the PAST Semester, were you ever in a situation where your teacher…?**			
Teacher Incivility	CIAT1	The teacher told me off without listening to me.	0.71
Cronbach’s alpha (“α”) = 0.828	CIAT2	The teacher made fun of us and humiliated us.	0.68
CR = 0.913	CIAT3	Addressed me in unprofessional terms, either in class or publicly.	0.75
AVE = 0.568	CIAT4	Made insulting or disrespectful remarks during the class towards me.	0.89
	CIAT5	Will not meet with students outside the class when they needed.	0.71
	CIAT6	Make sexual remarks towards students/flirted with them.	0.75
	CIAT7	When the teacher was mad at a student, s/he punished the entire class	0.80
	CIAT8	The teacher did not encourage students to ask questions.	0.72
	CIAT9 *	The teacher showed rude behavior	0.59
**During the PAST YEAR, were you ever in a situation where, due to negative behavior and negative interaction with your teacher...?**			
Educational Effects	SEO1	It lowered my morale for active learning.	0.94
Cronbach’s alpha (“α”) = 0.841	SEO2	If I could, I would miss my lecture for this class.	0.88
CR = 0.934	SEO3	I get easily bored when taking a lecture during this class.	0.76
AVE = 0.739	SEO4	It lowered my final exam and class test performance.	0.85
	SEO5	It reduced my concentration and attention span to focus on the lecture.	0.86
**During the PAST YEAR, were you ever in a situation where, due to negative behavior and negative interaction with your teacher…?**			
Psychological Effects	PSYO1	I thought that l was unable to do things well as most students do.	0.72
Cronbach’s alpha (“α”) = 0.78	PSYO2	I could not concentrate on my study.	0.86
CR = 0.834	PSYO3	I thought of ways of taking revenge for my suffering.	0.71
AVE = 0.560	PSYO4	I had the impression that I am not good at anything.	0.69

* Items were dropped; CIAT, Conflict-Inducing Attitude; SEO, Students Educational Outcomes; PSYO, Psychological Outcomes.

**Table 4:** Correlation matrices for path analysis.

Variables	Mean	SD	1	2	3	4	5	6
1. Incivility	3.14	0.84	(0.75)				
2. Discrimination	3.27	0.82	0.27 **	(0.74)				
3. University Resources	3.52	0.89	0.20 **	0.58 **	(0.76)			
4. Conflict-Inducing Attitude	3.30	0.79	0.18 **	0.43 ***	0.39 **	(0.75)		
5. Educational Outcomes	3.57	0.88	0.04	0.31 **	0.36 **	0.37 **	(0.86)	
6. Psychological Outcomes	3.50	0.84	−0.06	0.07	0.59 **	0.63 **	0.60 **	(0.75)

Note: The square root of average variance extracted presented within parenthesis. Correlation is significant at the level ** *p* < 0.01; *** *p* < 0.001; two-tailed test.

**Table 5:** Path analysis parameter estimates, their standard errors, and their significance.

Variables	*β*-Value	*t*-Value	*p*-Value	Error (SE)	Decision
H1: (TI—CIAT)	0.605	11.783	0.001	0.051	Supported
H2: (TD—CIAT)	0.092	1.454	0.147	0.063	Not Supported
H3: (UR—CIAT)	0.206	3.559	0.001	0.058	Supported
H4: (CIAT—SEO)	0.211	2.980	0.004	0.071	Supported
H5: (CIAT—PSYO)	0.845	20.038	0.001	0.042	Supported

TI, Teacher Incivility; TD, Teacher Discrimination; UR, University Resources; SEO, Students Educational Outcomes; CIAT, Conflict-Inducing Attitude; PSYO, Psychological Outcomes; Standardized estimation shown, *N* = 130.
